# A new approach combining a whole-slide foundation model and gradient boosting for predicting BRAF mutation status in dermatopathology

**DOI:** 10.1016/j.csbj.2025.06.017

**Published:** 2025-06-06

**Authors:** Mohamed Albahri, Daniel Sauter, Felix Nensa, Georg Lodde, Elisabeth Livingstone, Dirk Schadendorf, Markus Kukuk

**Affiliations:** a Department of Computer Science, Dortmund University of Applied Sciences and Arts, Dortmund 44227, Germany; b Department of Dermatology, University Hospital Essen, Essen 45147, Germany; c Institute for AI in Medicine (IKIM), University Hospital Essen, Essen 45131, Germany; d Institute of Diagnostic and Interventional Radiology and Neuroradiology, University Hospital Essen, Essen 45147, Germany

**Keywords:** Melanoma, Deep learning, Foundation model, Machine learning, Whole slide image, Computational pathology

## Abstract

Determining the mutation status of proto-oncogene B-Rapidly Accelerated Fibrosarcoma (BRAF) is crucial in melanoma for guiding targeted therapies and improving patient outcomes. While genetic testing has become more accessible, histopathological examination remains central to routine diagnostics, and an image-based strategy could further streamline the associated time and cost. In this study, we propose a new machine learning framework that integrates a large-scale, pretrained foundation model (Prov-GigaPath) with a gradient-boosting classifier (XGBoost) to predict BRAF-V600 mutation status directly from histopathological slides. Our approach was trained and cross-validated on the Skin Cutaneous Melanoma (SKCM) dataset from The Cancer Genome Atlas (TCGA; 275 slides), where the fine-tuned Prov-GigaPath model alone achieved an average Area Under the Curve (AUC) of 0.653 during cross-validation. An additional test on 68 slides from the University Hospital Essen (UHE), Germany, yielded an AUC of 0.697 (95 % CI: 0.553–0.821). Incorporating XGBoost significantly improved performance, reaching an AUC of 0.824 (SD=0.043) during cross-validation and 0.772 (95 % CI: 0.650–0.886) on the independent set—representing a new state-of-the-art for image-only BRAF mutation prediction in melanoma. By employing a weakly supervised, data-efficient pipeline, this method reduces the need for extensive annotations and costly molecular assays. While these results are not intended to replace genetic testing at this stage, they mark a new milestone in predicting BRAF mutation status solely from histopathological slides—a concept not yet fully established in prior research—and underscore the potential for seamlessly integrating automated, AI-driven decision-support tools into diagnostic workflows, thereby expediting personalized therapy decisions and advancing precision oncology.

## Highlights

• New weakly supervised, image-only pipeline for BRAF-V600 mutation detection.
• State-of-the-art AUCs on TCGA and independent UHE cohort without manual annotations.
• Data-efficient and reproducible on small, heterogeneous datasets; code and methods publicly released.
• Systematic comparison of multiple foundation encoders with RF, LR, and XGBoost classifiers.
• Framework for scalable digital-pathology workflows and future multimodal precision-oncology studies.

## Graphical Abstract

**Figure:**
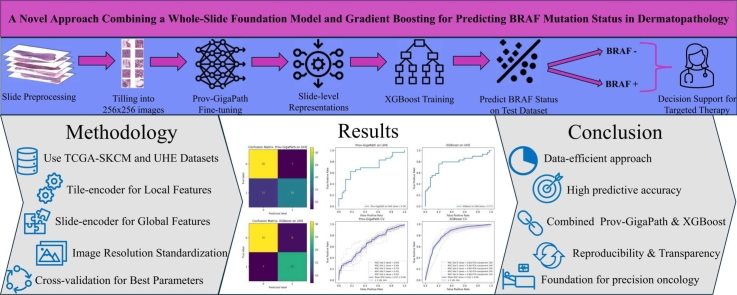

## 1 Introduction

Melanoma is a highly aggressive form of skin cancer that accounts for a significant proportion of skin cancer-related mortality worldwide. Therefore, precise characterization of melanoma is essential for effective treatment and improving patient outcomes [1]. Among the genetic mutations associated with melanoma, BRAF mutations —particularly the V600 variant—hold particular significance. BRAF is one of the most frequently mutated genes in melanoma, with the V600E mutation being the most prevalent variant, occurring in approximately 50 % of cases [2]. This mutation leads to the constitutive activation of the BRAF/MEK/ERK signaling pathway, driving tumor growth and survival [3], [4]. Targeted therapies using the combined BRAF/MEK inhibitors have been shown to improve survival outcomes in patients harboring the BRAF V600E mutation [5]. Consequently, molecular pathological examination is essential for the precise detection of potential BRAF driver mutations and for developing optimal treatment strategies for melanoma patients [6], [7].

In parallel, the concept of foundation models—initially popularized by language models trained on vast corpora—has since expanded to other modalities such as images, music, and more. In medical imaging and diagnostics, these models, pre-trained on extensive datasets, have demonstrated remarkable versatility. By fine-tuning these models for specific tasks, they offer state-of-the-art performance in complex classification problems, significantly advancing the field [8]. In digital pathology, foundation models such as Prov-GigaPath [9] have demonstrated significant potential in analyzing histopathological images, extracting relevant features, and providing accurate predictions. The widespread adoption of these models is transforming clinical practices by enabling more precise and efficient diagnostics [10], [11].

In cases where sample data can be effectively represented as tabular data in the form of feature vectors, traditional machine learning (ML) methods like decision trees or gradient boosting have been shown to perform well, particularly when working with limited datasets [12], [13]. These algorithms excel at making accurate predictions in low-dimensional spaces and can be more efficient than deep learning models, which often require large amounts of data for optimal performance [12]. Traditional ML models are also well-suited for scenarios where the input data can be structured into interpretable features, such as when complex data like histopathological images are preprocessed into numerical representations. Studies have demonstrated that ML techniques can outperform deep learning models when training data is scarce, as they tend to overfit less and can leverage feature selection to enhance generalizability [12], [13].

EXtreme Gradient Boosting (XGBoost) [14], an efficient implementation of the gradient boosting algorithm [15], is widely recognized for its performance and reliability in classification tasks. It excels in handling structured data and provides robust solutions by integrating multiple weak learners to create a strong predictive model. The combination of foundation models with XGBoost can synergistically harness the strengths of both approaches, leading to enhanced performance in predictive tasks.

### 1.1 Study objective

In this study, we address the challenge of predicting BRAF-V600 mutation status in melanoma by employing a new approach that integrates the Prov-GigaPath foundation model with an XGBoost classifier. Our method focuses on image-based classification, utilizing the advanced capabilities of the tile encoder and slide encoder components of the Prov-GigaPath model [9]. We fine-tuned the Prov-GigaPath model to generate slide-level representations, which were then used to train an XGBoost classifier for enhanced predictive accuracy. The model was trained on the TCGA dataset [16], [17] (a publicly accessible and diverse collection of histopathological images). For validation, we applied a cross-validation technique and tested the model on the independent UHE dataset.

To further validate the generalizability of our pipeline, we benchmarked Prov-GigaPath against three other publicly available foundation models (CONCH v1.5 [18], Virchow [11], and CTransPath [19])—each paired with its recommended slide encoder—and evaluated all combinations under identical 5-fold cross-validation and external testing protocols [20] using three downstream classifiers (XGBoost [14], Random Forest [21], and Logistic Regression [22]) on PCA-reduced embeddings.

### 1.2 Motivation

Although advancements in sequencing technologies have made genetic testing faster and more accessible, there are several compelling reasons to pursue image-based prediction of BRAF status [4]. First, histopathological analysis of tumor samples remains a standard practice in clinical settings, and the ability to predict BRAF status directly from these images could streamline diagnostics, reducing the need for additional molecular tests. Second, image-based methods can be particularly valuable in low-resource settings where access to advanced sequencing technologies may be limited [23]. Furthermore, image-based prediction can provide immediate feedback during biopsy examination, potentially guiding real-time clinical decisions [4].

### 1.3 Contributions

Our research offers several key contributions:

1) **New Weakly Supervised Image-Only Method:** We introduce a new approach that combines the Prov-GigaPath foundation model and XGBoost to identify BRAF mutation status solely from standard histopathological slides, reducing dependence on extensive manual annotations and molecular assays.
2) **Validated the Feasibility of Morphology-Based BRAF Mutation Status Prediction with State-of-the-Art Performance:** Through extensive experimentation and cross-validation on the public TCGA dataset—and further testing on an independent set from the University Hospital Essen—we highlight the viability of morphology-based BRAF mutation status prediction in melanoma, achieving state-of-the-art results in image-only classification.
3) **Demonstrated Data Efficiency and Reproducibility:** Our approach showcases robust performance despite operating on relatively small and heterogeneous datasets. By releasing both our code and methodologies, we promote transparency and facilitate further developments in melanoma diagnostics.
4) **Comprehensive Model Comparison and Validation:** We systematically evaluated three additional foundation models (CONCH/TITAN, Virchow/PRISM, and CTransPath/CHIEF), both as end-to-end classifiers and as feature extractors combined with Random Forest, Logistic Regression, and XGBoost.
5) **Foundation for Future Studies**: By integrating a powerful foundation model with a traditional ML algorithm, this work underscores the potential for scalable and efficient pipelines in digital pathology, paving the way for the incorporation of multimodal data, larger patient cohorts, and refined training techniques.

The significance of our study lies in advancing image-only classification methods for predicting BRAF mutation status in melanoma - a challenging and still largely unexplored area in the field. By integrating the Prov-GigaPath foundation model with the robust XGBoost algorithm, our approach demonstrates a reliable, scalable, and reproducible strategy for this critical diagnostic task. This contribution not only offers compelling evidence that image-based prediction of BRAF mutation status is potentially feasible—a concept that has not been sufficiently proven in prior research—but also highlights the data-efficient nature of our methodology. By leveraging a relatively small yet challenging dataset, our study underscores the potential of accessible and reproducible approaches in advancing melanoma diagnostics and guiding targeted therapies, ultimately contributing to the field of precision oncology.

## 2 Related work

The prediction of BRAF mutation status in melanoma has garnered significant attention due to its implications for targeted therapy and patient management. Several notable studies have contributed to this field, employing various methodologies and datasets.

Schneider et al.[24] conducted a study using the TCGA dataset to develop a multimodal model integrating image, clinical, and methylation data. Their approach demonstrated strong performance, underscoring the advantages of multimodal integration. For the image-only component, they used a random forest classifier [21] on features extracted from H&E-stained slides. These slides were tessellated into 256 × 256 pixel patches, with a feature extractor trained on the Camelyon17 [25] dataset producing 512-dimensional feature vectors. Tumor regions were manually annotated, followed by standard H&E staining and preprocessing. Despite these efforts, their image classifier achieved an AUC of only 0.64 on the TCGA dataset, highlighting the challenges in purely image-based prediction for BRAF mutation status.

Recent studies have also explored the use of multiple instance learning and self-supervised learning (SSL) models trained on histopathological images for prognostic biomarker discovery. For instance, Godson et al. [26] developed SSL-based MIL models to classify melanoma whole-slide images into immune subtypes, demonstrating the growing relevance of domain-specific SSL approaches in pathology. Their work illustrates the potential of pathology-trained SSL feature extractors in learning biologically meaningful representations from routine H&E images without requiring additional molecular profiling. This trend supports broader efforts to advance image-based precision medicine through weakly supervised deep learning methods.

Kim et al. [23] fine-tuned a model using manually annotated whole slide images (WSIs). Their approach involved a five-fold cross-validation, training an Inception v3 model at different magnifications (5x, 10x, and 20x). They explored both transfer learning and training all layers from scratch. The model trained from scratch achieved an AUC of 0.71 (SD=0.06) for the New York University (NYU) cohort and 0.67 (SD=0.08) for the TCGA cohort. When applying transfer learning, the performance improved to an AUC of 0.77 (SD=0.10) for the NYU cohort, while the TCGA performance remained at 0.67 (SD=0.06). Notably, their study relied on detailed manual annotations for WSIs and was tested on a smaller subset of 28 TCGA WSIs.

Vorontsov et al. [4] introduced the Virchow foundation model, trained on approximately 1.5 million WSIs. The model achieved state-of-the-art results across various tasks, including BRAF mutation status prediction in melanoma, with an AUC of 0.827 on their test dataset. They also reported that the UNI model [27] achieved similar results (AUC=0.823) on their test dataset. For BRAF prediction in melanoma specifically, the Virchow model was trained and tested on a non-public dataset comprising 868 slides for training and 138 slides for testing. The Virchow model was trained and tested on a larger, non-public dataset, which limits direct comparisons with other works. Additionally, while the tile-encoder component of the Virchow model was available upon request, the complete codebase was not publicly accessible at the time of the study [4], complicating the reproduction and application of their approach to public datasets like TCGA.

Prov-GigaPath [9] is a state-of-the-art whole-slide pathology foundation model that excels in 25 out of 26 tasks, including 9 cancer subtyping and 17 pathomics tasks, pretrained on 1.3 billion image tiles from over 171,000 whole slides, covering 31 major tissue types. It combines a tile encoder for capturing local features and a slide encoder for integrating global features using the LongNet method [9], [28]. In our study, we leveraged the Prov-GigaPath model to generate slide-level representations for predicting BRAF mutation status in melanoma. This model's ability to effectively capture both local and global pathological features makes it a robust foundation for our image-only classification approach. Further details on the fine-tuning and integration with the XGBoost classifier are provided in the Materials and Methods section.

In summary, while significant progress has been made in predicting BRAF mutation status in melanoma, many approaches rely on multimodal data, require detailed manual annotations, or depend on very large datasets. The potential of data-efficient, image-only methods, especially in a weakly-supervised setting, remains underexplored.

## 3 Materials and methods

### 3.1 Datasets

We utilized the TCGA dataset [16], [17] for training and cross-validation. This dataset originally contains 475 slides, but BRAF mutation status is available for only 276 of these slides. One slide was excluded after automated foreground/background segmentation (using a single, dataset-wide threshold) failed to yield a valid tissue mask. Manual inspection revealed that this slide exhibited excessive blurring and pixelation relative to the remainder of the cohort, justifying its removal for insufficient image quality. Consequently, 275H&E-stained WSIs—comprising both primary and metastatic melanomas with confirmed BRAF status—were retained for analysis. The TCGA repository is publicly accessible and provides a heterogeneous, large-scale compendium of WSIs.

**Fig. 1:**
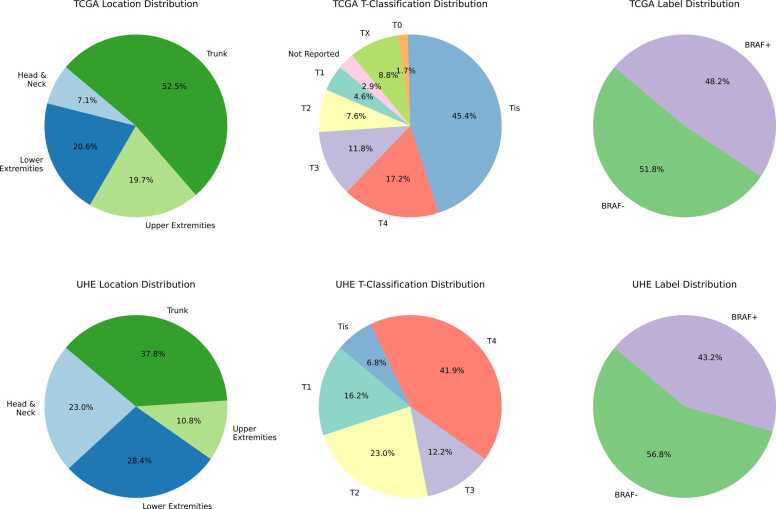
**Distribution of clinical features across the TCGA and UHE datasets**: The first column shows the location distributions for TCGA and UHE datasets, followed by the T category classifications and BRAF mutation status distributions. The T classification (T0-T4, TX, Tis) reflects the primary tumor size and extent of spread, a critical factor in staging melanoma and guiding treatment decisions. The more advanced classifications (e.g., T3 and T4) typically indicate larger and more invasive tumors, influencing both prognosis and therapeutic strategy [16].

For independent testing, we used the UHE dataset, a proprietary collection comprising 268 slides, with BRAF mutation status available for 74 slides of primary melanomas. Six slides were automatically excluded from the dataset because tissue areas could not be detected using the unified threshold applied for segmentation across all slides, as we aimed to maintain a fully automated approach without adjusting thresholds for individual slides This resulted in 68 slides being included in the final analysis. Fig. 1 shows the Distribution of clinical features across the TCGA and UHE datasets.

The ground truth labels for BRAF mutation status were obtained from genome sequencing files for the TCGA dataset, and from patient records for the UHE dataset.

### 3.2 Prov-GigaPath Foundation Model for Slide-Level Representation Learning

Our approach utilizes the Prov-GigaPath foundation model [9], which takes image tiles from a pathology slide as input and outputs slide-level representations for diverse clinical applications. Prov-GigaPath excels in long-context modeling of gigapixel pathology slides by distilling varied local pathological structures and integrating global signatures across the whole slide [9].

**Fig. 2:**
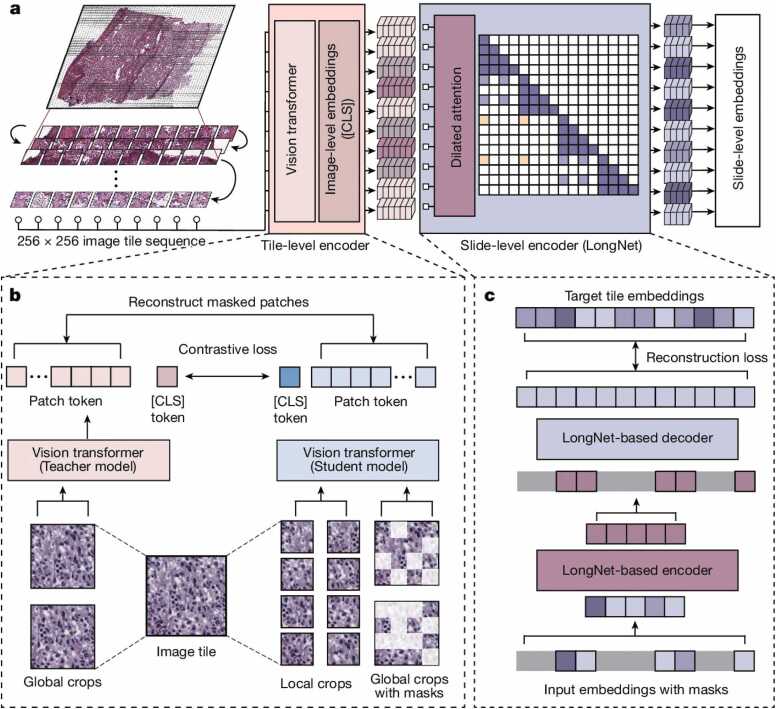
**Overview of Prov-GigaPath**[9]: **a**) Flow chart illustrating the Prov-GigaPath model architecture. Each input WSI is divided into a sequence of 256 × 256 image tiles in row-major order, which are then processed by an image tile-level encoder to generate visual embeddings. These embeddings are subsequently processed by a slide-level encoder based on the LongNet architecture to create contextualized embeddings for various downstream applications. **b**) Pretraining process for image tiles using the DINOv2 framework. **c**) Slide-level pretraining using LongNet with a masked autoencoder approach. The classification token [CLS] is used to aggregate information for classification tasks. Reproduced from [9] under the terms of the Creative Commons Attribution 4.0 International (CC BY 4.0) license: http://creativecommons.org/licenses/by/4.0/.

Prov-GigaPath consists of two main components (see Fig. 2):

• **Tile Encoder**: Captures local features by individually projecting all tiles into compact embeddings. It is pretrained using DINOv2 [29], a state-of-the-art image self-supervised learning framework.
• **Slide Encoder**: Captures global features by inputting the sequence of tile embeddings and generating contextualized embeddings using a transformer. It combines masked autoencoder pretraining with LongNet [28], a method for ultra long-sequence modeling.

For downstream tasks, the slide encoder's output is aggregated using a softmax attention layer [9]. Prov-GigaPath is pretrained on large and diverse real-world data, which can be fine-tuned for specific tasks.

In our study, we fine-tuned the pretrained slide encoder of Prov-GigaPath to generate slide-level representations. These representations were then used to train an XGBoost classifier, ensuring efficient feature extraction and robust classification performance.

### 3.3 Preprocessing and Fine-Tuning

**Fig. 3:**
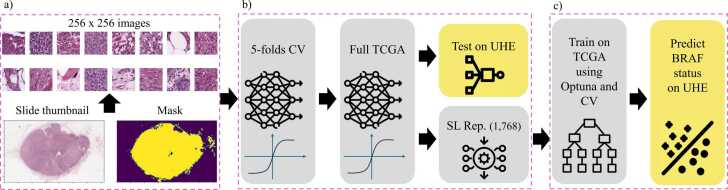
**Workflow for Model Training and Testing in BRAF Status Prediction: a)** Preprocessing Steps: The slides are divided into patches sized according to the resolution used for the Prov-GigaPath model. **b)** 5-Folds Cross-Validation (CV) and Full TCGA Dataset Training: This section shows the initial steps where the Prov-GigaPath model is trained using 5-fold cross-validation on the TCGA dataset, followed by training on the entire TCGA dataset without validation or test splits, to predict and create slide-level representations (SL). **c)** Optimization and Final Prediction: The final section demonstrates the use of Optuna and cross-validation on the TCGA dataset to find the best parameters for training the XGBoost classifier. This optimized classifier is then employed to predict BRAF status on the UHE test dataset.

Given the enormous size of WSIs, which can reach up to 4 GB, it is impractical to use entire slides directly for training [30], [31]. Therefore, a tiling approach was employed to process the slides efficiently. Fig. 3 shows the workflow we used in this study.

#### 3.3.1 Slide-Level Tiling and Foreground Segmentation

To manage the large image sizes, we first separated the foreground from the background using a threshold value of 0.8 after applying a Gaussian filter with a sigma value of 10 to the thumbnail image of the WSI. The Gaussian filter with a sigma of 10 was chosen because it effectively smooths the image, reducing noise and minor variations while preserving essential tissue structures. This level of smoothing facilitates a clear distinction between the tissue regions and the background. The threshold value of 0.8 was selected based on preliminary experiments and standard practices in histopathological image analysis, ensuring that all relevant tissue areas are included while excluding background regions. This combination of parameters proved effective for our dataset, successfully isolating the tissue for further analysis. This step is crucial as it focuses computational resources on the pertinent regions of the slide, enhancing the efficiency and accuracy of subsequent processing steps. An example of the resulting mask is shown in Fig. 3. Similar methodologies have been utilized in previous studies, such as [32].

#### 3.3.2 Normalizing Slide Resolution

Since not all slides have the required resolution of 0.5 micrometers per pixel (mpp), which is essential for the Prov-GigaPath model [9], we standardized the resolution by tiling the slides based on a target patch size. This was achieved using the formula:

(1) $$S={\lfloor P}_{t}\times \frac{M_{t}}{R_{0}}\rfloor \;$$

Where S is the resulted patch size, ​​​​​​$P_{t}$ is the target patch size, $M_{t}$ is the target *mpp* and $R_{0}$ is the *mpp* at level zero. This approach compensates for slides that do not have the target resolution (mpp=0.5) directly [27].

#### 3.3.3 Patch Extraction and Filtering

The x-y coordinates of the upper left corner of each patch, along with the calculated patch size, were saved. During inference, these coordinates were used to load patches dynamically from the WSI, avoiding the need to save them on disk, which would require excessive memory. Patches were first converted to grayscale and then filtered to exclude patches with more than 80 % white space, such as patches of fat tissue [23]. The remaining patches were then stain normalized using the Macenko method [33] via the staintools package [34] and resized to the default input image size required by Prov-GigaPath, which is 256 × 256.

#### 3.3.4 Extracting Tile Embeddings from the Prov-GigaPath Tile Encoder

The tile encoder of the Prov-GigaPath model processed these patches, and the resulting embeddings with dimensions of 1 by 768, were saved. Each WSI was represented by a file containing the corresponding tile embeddings, which were subsequently used as input for the slide encoder.

#### 3.3.5 Fine-Tuning the Prov-GigaPath Slide Encoder and Training Data Split

The pretrained slide encoder was employed in unfrozen mode for fine-tuning to predict BRAF mutation status and later switched to evaluation mode to generate slide-level representations. For prediction, we appended a final linear layer to the model and fine-tuned the slide encoder on the TCGA dataset using the AdamW optimizer [35] with a base learning rate of 0.003 for 5 epochs. Due to the slide encoder's limitation of processing only one slide at a time [9], we employed gradient accumulation to simulate a batch size of 32. The selection of the AdamW optimizer, learning rate, and number of epochs was guided by their effectiveness in handling weight decay, ensuring stable convergence, and preventing overfitting, as recommended in prior studies [4], [35] and consistent with the settings used in training the Prov-GigaPath model [9].

The TCGA dataset was initially divided in a similar way to the division used for the Prov-GigaPath model, with 60 % for training, 30 % for testing, and 10 % for validation [9]. To identify the optimal parameters for fine-tuning, we experimented with different feature layers of the slide encoder. Through empirical testing, we found that focusing on the 9th feature layer [9] provided the best cross-validation results for predicting BRAF mutation status. This suggests that the 9th layer captures the most relevant features for our specific task. Therefore, we selected the 9th feature layer for fine-tuning and conducted 5-fold cross-validation. This approach ensured the model's robustness against potential splitting biases. During both the training and testing phases, all patches saved for each slide were utilized.

After identifying the optimal hyperparameters of the model on the TCGA dataset through cross-validation, the entire TCGA dataset was consolidated into a single training set for fine-tuning, without further subdivision into validation or test subsets. This decision was made because model selection for fine-tuning and then generating slide-level representations was based on the final training epoch rather than validation performance [9]. Therefore, a separate validation/test set was deemed unnecessary. The fine-tuning process employed the optimal configurations derived from cross-validation. This approach enabled the inclusion of the full TCGA dataset during training, including the 40 % previously held out for cross-validation and testing, thus significantly expanding the diversity and volume of training data. The objective was to refine the model’s ability to capture the distinct patterns associated with BRAF mutation status in melanoma by leveraging the increased data availability, which can increase the predictive performance of the model. At this stage, the independent test dataset had not yet been introduced to the model.

#### 3.3.6 Evaluating Prov-GigaPath’s Predictive Performance

After fine-tuning on the full TCGA dataset, we tested the Prov-GigaPath model's ability to predict BRAF status on the UHE test dataset for the first time in evaluation mode, demonstrating its strength in predicting biomarkers consistent with the authors' claims regarding Prov-GigaPath's excellence in biomarkers predictions [9]. The trained slide encoder model was in parallel used in evaluation mode to generate slide-level representations with the shape of (1, 768) for each slide [9]. These representations were generated for both the TCGA training and UHE test datasets and were subsequently used to train the XGBoost classifier.

#### 3.3.7 Training and Evaluating the XGBoost Classifier Using Slide-Level Representations

We utilized the slide-level representations generated for each slide in the TCGA dataset to train the XGBoost classifier. The TCGA dataset was split into 80 % for training and 20 % for validation. Initially, a StandardScaler [36] was applied to normalize the data, followed by dimensionality reduction using Principal Component Analysis (PCA) [36], [37] on the TCGA dataset. To prevent data leakage, the test dataset was transformed using the same StandardScaler and PCA parameters derived from the TCGA training data. For hyperparameter optimization, we employed Optuna [38], conducting 1000 iterations of parameter search, each involving 5-fold cross-validation [36] on the TCGA dataset.

Considering that the possible values for PCA components in our case range from 1 to a maximum of 220 (since we use 80 % of the 275 TCGA slides for training during cross-validation), we needed to select component values that effectively balance dimensionality reduction with information retention. Very small component values (e.g., below 30) would result in significant loss of variance captured from the original data, leading to overly sensitive dimensionality reduction and potential degradation of model performance. Conversely, values approaching the maximum (close to 220) offer minimal dimensionality reduction benefits and increase computational complexity without substantially improving performance.

To systematically cover the most important characteristics of the interval and capture a representative range of cumulative explained variance, we selected three PCA component values: 60, 100, and 170. These values correspond approximately to low, medium, and high levels of retained variance within the dataset, allowing us to explore the impact of dimensionality reduction at different scales. This strategic selection enabled us to focus our optimization efforts on key points along the dimensionality spectrum without exhaustively testing every possible value—a process that would have been computationally prohibitive given that each PCA value would require training the classifier at least 5000 times (1000 hyperparameter optimization iterations × 5-fold cross-validation).

Furthermore, since PCA serves as a preprocessing step, our goal was to ensure sufficient variance capture for effective classification rather than optimizing the entire model solely based on this step. Even if we tested more values, similar questions about the choice of specific component numbers would arise, necessitating a practical decision. Ultimately, the PCA component value of 100, in conjunction with the optimal XGBoost parameters found using Optuna, yielded the highest AUC on the TCGA dataset and was therefore selected. The resulting classifier, trained with these best-performing parameters, was then used to predict BRAF mutation status on the UHE test dataset.

#### 3.3.8 Validation Experiments

To rigorously validate our pipeline, we compared Prov-GigaPath against three additional publicly-available foundation models—CONCH v1.5 [18], Virchow [11], and CTransPath [19]—each paired with its recommended slide encoder (Titan [39], PRISM [40], and CHIEF [41], respectively). For that, all preprocessing (tissue segmentation, patching, stain normalization and feature extraction) was performed with Trident-Toolkit [20] to ensure an identical data-processing backbone.

1. End-to-End Slide Classification
  • Models: Prov-GigaPath; CONCH v1.5 + Titan; Virchow + PRISM; CTransPath + CHIEF.
  • Training: For each encoder we appended a one-layer classifier, fine-tuned on TCGA using 5-fold cross-validation (folds fixed) for 5 epochs with learning rate = 1e-3.
  • Evaluation: We report mean ± SD AUC across TCGA folds, then benchmark all four models on the held-out UHE cohort.
2. Feature-Extraction + Classical ML
  • Embeddings: Slide-level representations extracted via Trident.
  • Dimensionality Reduction: PCA with n_components ∈ {60, 100, 170}.
  • Classifiers und Hyperparameter Search: XGBoost [14], Random Forest [21], and Logistic Regression [22] each tuned via 10-iteration randomized search under 5-fold cross-validation on TCGA.
  • External Test: Final models retrained on full TCGA set and evaluated on UHE.

By holding all non-model factors constant (data splits, preprocessing, optimization schedule), this validation framework isolates the comparative strengths of each foundation–classifier combination.

## 4 Results

**Fig. 4:**
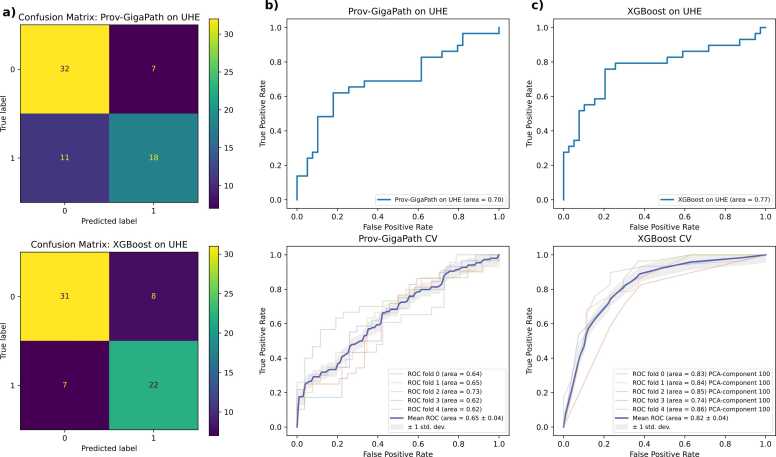
**Performance metrics visualization for the Prov-GigaPath model and the XGBoost classifier**: **a)** Confusion matrices on the UHE test set. **b)** ROC curves for the Prov-GigaPath model on the UHE test set and on the TCGA dataset using cross-validation folds. **c)** ROC curves for the XGBoost classifier on the UHE test set and on the TCGA dataset using cross-validation folds. Shaded areas represent standard deviation across folds. (UHE: University Hospital Essen; CV: Cross-Validation; PCA: Principal Component Analysis).

Our study involved two key phases: fine-tuning the Prov-GigaPath model and training an XGBoost classifier using the resulting slide-level representations. The outcomes of these phases are presented in Table 1 and illustrated in Fig. 4.

**Table 1:** **Performance Metrics for Prov-GigaPath Model and XGBoost Classifier**: This table presents the results of the Prov-GigaPath model and XGBoost classifier applied to the TCGA training dataset and the independent UHE test dataset. The metrics include the 95 % confidence intervals (CI) estimated with bootstrap for the UHE test dataset and standard deviations (SD) across cross-validation (CV) folds on the TCGA dataset. For XGBoost we used pca-component= 100.

**Model**	**Dataset**	**AUC (±SD)**	**Acc (±SD)**	**Prec (±SD)**	**Rec (±SD)**	**F1 (±SD)**	**Sens**	**Spec**
**GigaPath**	TCGA	0.653 ± 0.039	0.611 ± 0.048	0.579 ± 0.066	0.93 ± 0.068	0.708 ± 0.04	--	--
	UHE	0.697 [0.553,0.821]	0.735	0.72	0.62	0.667	0.621	0.821
**GigaPath + XGBoost**	TCGA	**0.824** **± 0.043**	0.77 ± 0.039	0.74 ± 0.039	0.87 ± 0.052	0.80 ± 0.035	--	--
	UHE	**0.77 [0.650,0.886]**	0.78	0.77	0.78	0.78	0.759	0.795

(AUC = Area Under the ROC Curve; Acc = Accuracy; Prec = Precision; Rec = Recall; F1 = F1-Score; Sens = Sensitivity; Spec = Specificity; SD = Standard Deviation)

### 4.1 Phase 1: Fine-Tuning and Evaluating the Prov-GigaPath Model

Our primary objective in the first phase was to fine-tune the Prov-GigaPath model to generate robust slide-level representations suitable for downstream classification tasks. We employed 5-fold cross-validation on the TCGA dataset to determine the optimal parameters for fine-tuning. This process resulted in an average AUC of 0.653 ± 0.039, indicating moderate predictive ability based solely on the fine-tuned model. The mean accuracy was 0.611 ± 0.048, with a precision of 0.579 ± 0.066, recall of 0.93 ± 0.068, and F1-score of 0.708 ± 0.040.

While our main goal was to refine the model for feature extraction, we additionally tested its performance on the independent UHE test dataset to assess its predictive capability. The model achieved an AUC of 0.697 (95 % CI: [0.553, 0.821]). The accuracy was 0.735, with a precision of 0.72, recall of 0.62, F1-score of 0.667, sensitivity of 0.621, and specificity of 0.821. The confusion matrix (Fig. 4a, top) shows the model correctly predicted 32 negatives and 18 positives, suggesting a higher true negative rate.

### 4.2 Phase 2: Training and Evaluating the XGBoost Classifier

Following the generation of slide-level representations from the fine-tuned Prov-GigaPath model, we proceeded to train an XGBoost classifier to enhance predictive performance. The cross-validation process on the TCGA dataset yielded an average AUC of 0.824 ± 0.043, a significant improvement over the first phase. The mean accuracy was 0.77 ± 0.039, precision 0.74 ± 0.039, recall 0.87 ± 0.052, and F1-score 0.80 ± 0.035.

In the final evaluation on the independent UHE test dataset, the XGBoost classifier demonstrated strong predictive performance, achieving an AUC of 0.772 (95 % CI: [0.650, 0.886]). The accuracy was 0.78, with a precision of 0.77, recall of 0.78, F1-score of 0.78, sensitivity of 0.759, and specificity of 0.795. The confusion matrix (Fig. 4a, bottom) indicates balanced true positive and true negative rates, with 31 negatives and 22 positives correctly predicted.

To assess whether the observed AUC improvement when integrating XGBoost was statistically significant, we applied two tests. First, a paired Wilcoxon signed-rank test [42] on the five cross-validation AUCs (Prov-GigaPath vs. Prov-GigaPath + XGBoost) yielded W = 0 with a one-sided p = 0.062, indicating a consistent trend toward higher AUCs that narrowly missed the conventional 0.05 threshold. Second, on the independent UHE cohort (n = 68), DeLong’s test [43] comparing the two ROC curves produced z = –1.332 (two-sided p = 0.183; one-sided p = 0.092), again showing a favorable but non-significant difference in discrimination.

#### 4.2.1 Comparative Performance of Foundation Models as End-to-End Classifiers

We fine-tuned four slide encoders (Prov-GigaPath, CHIEF, PRISM and TITAN) by appending a single linear classification head to predict BRAF-V600 mutation status directly from whole-slide images. Five-fold cross-validation on the TCGA cohort yielded mean AUCs ranging from 0.559 to 0.664, with Prov-GigaPath achieving 0.653 ± 0.039 and TITAN the best result at 0.664 ± 0.093. On the independent UHE test set, all models attained AUCs between 0.617 and 0.697, demonstrating consistent—if moderate—discriminative power across architectures (Table 2).

**Table 2:** **End-to-end classification performance of foundation models:** End-to-end BRAF-V600 prediction performance (mean AUC ± SD) of fine-tuned slide-encoder models on TCGA (5-fold CV) and independent UHE test set.

Slide Encoder	Tile Encoder	CV-AUC (±SD)	CV-Acc (±SD)	CV-F1 (±SD)	Test AUC	Test ACC	Test F1
TITAN	Conch (v1.5)	**0.664 ± 0.093**	0.587 ± 0.092	0.573 ± 0.122	0.617	0.5	0.594
PRISM	Virchow	0.595 ± 0.086	0.601 ± 0.045	0.599 ± 0.075	0.645	0.516	0.591
CHIEF	Ctranspath	0.559 ± 0.059	0.529 ± 0.052	0.513 ± 0.101	0.612	0.616	0.206
GigaPath	Gigapath	0.653 ± 0.039	**0.611 ± 0.048**	**0.708 ± 0.04**	**0.697**	**0.735**	**0.667**

(AUC = Area Under the ROC Curve; Acc = Accuracy; F1 = F1-Score; CV = Cross-Validation; SD = Standard Deviation)

#### 4.2.2 Comparative Performance of Foundation Models as Feature Extractors + Traditional ML Classifiers

Next, we utilize each pretrained encoder to extract slide-level embeddings, applied PCA (n_components = 60, 100, 170), and trained three downstream classifiers (XGBoost, Random Forest, Logistic Regression) via a 10-iteration random search. In TCGA cross-validation, XGBoost consistently outperformed the others, with Prov-GigaPath + XGBoost reaching 0.792 ± 0.103 AUC. On the UHE test set, these feature-extraction + XGBoost pipelines maintained top performance (e.g. 0.751 AUC), confirming that classical ML on rich pretrained embeddings can substantially boost mutation-prediction accuracy. In Table 3, we report the results obtained with the optimal PCA setting (n_components=100).

**Table 3:** **Predictive performance of ML Classifiers:** The Predictive performance (AUC ± SD) of feature-extraction + ML pipelines (PCA n = 100; XGBoost, Random Forest, Logistic Regression) on TCGA (5-fold CV) and UHE test set.

Model	Classifier	CV-AUC (±SD)	CV-Acc (±SD)	CV-F1 (±SD)	Test AUC		Test ACC	Test F1
TITAN	RandomForest	0.695 ± 0.129	0.710 ± 0.090	0.737 ± 0.072		0.669	0.683	0.627
PRISM	RandomForest	0.568 ± 0.079	0.620 ± 0.069	0.638 ± 0.069		0.576	0.583	0.545
CHIEF	RandomForest	0.551 ± 0.079	0.597 ± 0.028	0.612 ± 0.073		0.477	0.5	0.482
GigaPath	RandomForest	0.605 ± 0.159	0.628 ± 0.080	0.638 ± 0.067		0.557	0.566	0.518
TITAN	LogisticRegression	0.692 ± 0.145	0.710 ± 0.101	0.716 ± 0.100		0.648	0.65	0.618
PRISM	LogisticRegression	0.597 ± 0.092	0.650 ± 0.090	0.677 ± 0.102		0.662	0.65	0.618
CHIEF	LogisticRegression	0.532 ± 0.067	0.605 ± 0.065	0.606 ± 0.113		0.515	0.55	0.470
GigaPath	LogisticRegression	0.557 ± 0.097	0.597 ± 0.074	0.585 ± 0.112		0.481	0.566	0.435
TITAN	XGBoost	0.661 ± 0.127	0.672 ± 0.063	0.694 ± 0.081		0.661	0.616	0.581
PRISM	XGBoost	0.542 ± 0.119	0.605 ± 0.098	0.609 ± 0.083		0.608	0.588	0.580
CHIEF	XGBoost	0.561 ± 0.103	0.582 ± 0.069	0.575 ± 0.074		0.446	0.566	0.524
GigaPath	XGBoost	**0.792 ± 0.103**	**0.781 ± 0.08**	**0.778 ± 0.05**		**0.751**	**0.71**	**0.72**

(AUC = Area Under the ROC Curve; Acc = Accuracy; F1 = F1-Score; CV = Cross-Validation; SD = Standard Deviation)

## 5 Discussion

This study aimed to develop and evaluate a robust, data-efficient approach for predicting BRAF mutation status in melanoma using the Prov-GigaPath model and an XGBoost classifier. Our results demonstrate that the integration of advanced deep learning models with classical machine learning techniques can yield competitive performance. However, there are several areas where our approach could be improved, and further research is needed to enhance its robustness and applicability.

### 5.1 Training and Evaluation of the Prov-GigaPath Model

The retraining of the Prov-GigaPath model was primarily driven by the need to adapt it specifically for predicting BRAF mutation status in melanoma, a task for which the original model was not explicitly designed. Initially, we employed the Prov-GigaPath model to generate slide-level representations and performed extensive cross-validation on the TCGA dataset to determine the optimal parameters for fine-tuning. This yielded an average cross-validation AUC of 0.653 (SD=0.0394), demonstrating the model’s potential but also highlighting the need for further refinement.

An initial evaluation using the independent UHE test dataset was conducted primarily to validate the model’s ability to generalize, rather than to optimize it for test performance. Following the fine-tuning process on the TCGA dataset, subsequent testing on the UHE dataset produced an improved AUC of 0.697. This enhancement in performance underscores the model’s ability to generalize effectively when trained on a larger and more diverse dataset. A key aspect of our approach was the decision to incorporate the entire TCGA dataset, including the 40 % previously reserved for cross-validation and testing, into the final training phase of Prov-GigaPath. This strategy allowed us to significantly increase the diversity and volume of training data, which proved crucial in enhancing the model’s predictive capabilities. The improved AUC on the test dataset validates our hypothesis that the model’s performance would benefit from this expanded dataset. The inclusion of more data enriched the model’s ability to capture the nuanced patterns associated with BRAF mutation status in melanoma.

Despite these improvements, the relatively modest AUC values indicate that while the model captures essential features, there remain complexities in the data that it does not fully account for. This suggests opportunities for further refinement and optimization. Overall, the findings confirm that increasing the dataset size, combined with targeted fine-tuning, can lead to meaningful performance gains in predictive modeling for this specific clinical task.

### 5.2 Training the XGBoost Classifier

The second phase involved using the slide-level representations generated by the Prov-GigaPath model to train an XGBoost classifier. This approach significantly improved performance, achieving an average AUC of 0.824 through cross-validation. The final evaluation on the UHE test dataset yielded an AUC of 0.772, demonstrating that the combination of deep learning for feature extraction and classical machine learning for classification can be highly effective.

We selected the XGBoost classifier due to its proven effectiveness on tabular data, which aligns with our slide-level representations (vectors of dimension 1 ×768). Given the relatively small size of our dataset, particularly at the slide representation level, we prioritized computational efficiency and model robustness by focusing on XGBoost. Established studies, such as Borisov et al. [13] and Shwartz-Ziv et al. [12], have demonstrated superior performance of XGBoost on tabular datasets, supporting our methodological choice. Additionally, initial experiments with a RandomForest classifier, following the approach of Schneider et al. [24], yielded suboptimal results. This reinforced our decision to utilize XGBoost, which has consistently shown enhanced predictive capabilities and robustness in comparable settings.

### 5.3 Comparison with Existing Methods

The closest study to ours, which used the TCGA dataset with cross-validation, was conducted by Schneider et al. [24]. Our approach improved the average AUC on the TCGA dataset by approximately 29 % (an absolute increase of 18.4 percentage points, from 0.64 to 0.824) compared to their image-only classifier.

While our results are promising, it is noteworthy that another study by Vorontsov et al. [4] reported a slightly higher AUC of 0.827 for predicting BRAF status in melanoma using the Virchow foundation model. This performance was achieved on a larger, non-public dataset, which included 868 slides for training and 138 slides for testing. Importantly, the full codebase and dataset from their study were not publicly available, making direct comparisons challenging. Given the similar performance reported with the UNI model, we also experimented with this model on the TCGA dataset. However, our cross-validation results^1^ yielded an AUC of approximately 0.60. Despite these efforts, direct comparisons between our study and that of Vorontsov et al. remain difficult due to the differences in datasets, experimental setups, and the non-public nature of their data. Nevertheless, it is important to note that, with only approximately 30 % of the slides used for training compared to the dataset employed by Vorontsov et al., our approach achieved comparable results. This outcome underscores the data efficiency of our method, demonstrating that our approach can achieve robust performance even with a relatively smaller dataset. The success of our approach in reaching similar performance metrics with fewer resources highlights its potential for practical applications where data may be limited, further reinforcing the validity and effectiveness of our methodology in predicting BRAF mutation status in melanoma. On the other hand, their findings reinforce our conclusion that BRAF mutation status in melanoma can be effectively detected from histopathological images, a concept that had not been fully established before.

In our head-to-head comparison of end-to-end slide classifiers, the TITAN encoder marginally outperformed all other architectures—including Prov-GigaPath—underscoring its strong built-in classification capacity. However, when leveraging foundation models purely as feature extractors and pairing them with traditional machine learning, the Prov-GigaPath + XGBoost pipeline achieved the highest accuracy and AUC across both cross-validation and the independent UHE test set. This result highlights Prov-GigaPath’s particularly effective slide representations for mutation prediction—a likely consequence of its intensive pretraining on biomarker-related tasks—and demonstrates that combining such rich embeddings with a robust classifier can yield superior predictive performance.

### 5.4 Limitations and Future Directions

Although our method demonstrates substantial potential for predicting BRAF mutation status, the current level of performance does not yet suffice to replace gold-standard molecular tests in clinical practice. One notable limitation of our study is the reliance on a single type of data modality (image data) for predicting BRAF mutation status. While our results demonstrate that image-only classifiers can be effective, integrating additional data modalities, such as clinical and genomic data, could further enhance predictive performance. Schneider et al. [24] showed that multimodal approaches could significantly improve performance, suggesting that future work should explore the integration of multiple data types.

We also employed a relatively simple threshold method to segment the foreground tissue and filter out fat tissues. Although this strategy proved functional, it requires manual parameter adjustments that may introduce variability and constrain reproducibility across diverse datasets. A more advanced segmentation approach, such as deep learning–based tissue detection, could yield more consistent and automated results.

Additionally, the relatively small size and limited diversity of both our training and test datasets present important limitations. In particular, a DeLong comparison of AUCs on the independent UHE cohort (n = 68) failed to reach statistical significance (two-sided p = 0.183), likely reflecting insufficient power rather than true equivalence. We therefore complemented this analysis with a paired Wilcoxon signed-rank test on the five cross-validation AUCs, which showed a consistent directional improvement with XGBoost (W = 0, one-sided p = 0.062) but again did not meet the conventional significance threshold. Together, these results suggest a real performance gain, yet underscore the need for larger, more homogeneous cohorts to validate and robustly quantify this effect.

Increasing the size and diversity of the datasets would not only strengthen these statistical comparisons, but also enhance the model’s robustness and generalizability by capturing a broader spectrum of clinical variability. Furthermore, leveraging more advanced foundation models pretrained on larger and more diverse biomarker datasets—potentially in combination with XGBoost—could substantially boost predictive performance. Recent advancements in slide representation techniques [44], which have demonstrated significant effectiveness in analyzing whole-slide images, may also further optimize model accuracy.

To ensure that this method becomes practically valuable for decision-makers, future research should focus on validating the approach in larger, multicenter studies. Collaborations with clinicians and healthcare stakeholders will be pivotal for tailoring the method to real-world needs and embedding it into standard diagnostic workflows. Finally, attention to regulatory and ethical considerations, as well as compliance with medical standards, will be essential steps toward broad clinical adoption.

## 6 Study significance and conclusion

This study advances melanoma diagnostics and BRAF mutation prediction by employing a data-efficient framework that integrates the Prov-GigaPath foundation model with a gradient-boosting (XGBoost) classifier. We first leveraged a weakly supervised pipeline on the publicly available TCGA dataset and validated our findings on an independent University Hospital Essen cohort, demonstrating robust predictive accuracy without the need for extensive manual annotations. To further validate the generality of our approach, we compared Prov-GigaPath against three other state-of-the-art patch/slide encoders (CONCH v1.5/TITAN, Virchow/PRISM, and CTransPath/CHIEF)—each fine-tuned under identical settings—and evaluated their embeddings with multiple traditional classifiers (XGBoost, Random Forest, and Logistic Regression) across PCA-reduced features. Although current performance does not yet support direct replacement of molecular testing in clinical practice, our results underscore the scalability and effectiveness of combining deep-learning–based morphology analysis with classical machine learning, enabling automated classification and guiding targeted therapies across hospitals and care settings. This work highlights the importance of reproducibility, transparency, and data efficiency in medical research and provides a solid foundation for future studies that incorporate multimodal data, larger datasets, and advanced training techniques. By making our code and methodologies publicly available, we aim to advance both the understanding and practical application of image-based mutation prediction in melanoma, paving the way for refinements in precision oncology and more efficient diagnostic workflows.

## Ethics Approval

The use of the UHE dataset was approved by the ethics committee at the University Hospital Essen under the approval number [No. 24-12073-BO, Year 2024]. All patient data used in this study was anonymized, and the research was conducted in compliance with ethical standards, including obtaining appropriate informed consent where applicable. No patient-identifiable information was used in the analysis, ensuring confidentiality and compliance with ethical guidelines.

## Funding

This research was supported by a PhD grant from the DFG Research Training Group 2535, "Knowledge- and data-based personalization of medicine at the point of care (WisPerMed)," at the University of Duisburg–Essen, Germany.

## CRediT authorship contribution statement

**Mohamed Albahri:** Writing – review & editing, Writing – original draft, Visualization, Validation, Software, Methodology, Data curation, Conceptualization. **Daniel Sauter:** Writing – review & editing, Data curation. **Felix Nensa:** Writing – review & editing, Supervision, Investigation. **Georg Lodde:** Writing – review & editing, Supervision, Investigation. **Elisabeth Livingstone:** Writing – review & editing, Supervision, Investigation. **Dirk Schadendorf:** Supervision, Investigation. **Markus Kukuk:** Writing – review & editing, Writing – original draft, Supervision, Investigation, Conceptualization.

## Data and Code Availability

The TCGA dataset used for training and cross-validation is publicly accessible at (https://portal.gdc.cancer.gov), and all relevant links and publications are cited in the manuscript. Due to ethical considerations, the UHE test dataset cannot be publicly shared.

All code developed in this study will be made publicly available at: (https://github.com/MohAlbahr/BRAF-Prediction).

## Originality and Submission

The manuscript is original, has not been published previously, and is not under consideration elsewhere.

## Author Approval

We confirm that the manuscript has been read and approved by all named authors and that there are no other persons who satisfied the criteria for authorship but are not listed. We further confirm that the order of authors listed in the manuscript has been approved by all of us. All Authors agree to the submission of the manuscript to Computational and Structural Biotechnology Journal.

## Declaration of Generative AI and AI-assisted technologies in the writing process

During the preparation of this work, the author(s) used OpenAI's ChatGPT to improve the readability and language of the manuscript. The tool was applied with human oversight, and the author(s) reviewed and edited the content as needed, taking full responsibility for the content of the published article. No AI-generated text was used to create research content or analytical findings.

## Declaration of Competing Interest

None.

## Figures and Tables

**Table B.1:** Best Hyperparameters for XGBoost Classifier

**Parameter**	**Value**
colsample_bytree	0.6
learning_rate	0.0005
max_depth	4
min_child_weight	8
n_estimators	800
subsample	0.4
reg_alpha	14.0
reg_lambda	8.0
scale_pos_weight	1.845

## Appendix A

To handle the computational demands of processing large whole-slide images (WSIs) and training deep learning models, we utilized the following resources:

*Hardware Configuration:*

• 2 x NVIDIA RTX 6000 GPUs, with 1024 GB RAM: For accelerated deep learning computations.
• 2 x AMD CPUs: For general processing tasks.

*Software Environment:*

• Programming Language: Python 3.9

*Libraries and Packages:*

• OpenSlide 3.4.1: WSI handling.
• scikit-learn 1.5.1: Machine learning utilities.
• huggingface-hub 0.20.2: Model management.
• timm > =1.0.3: Pretrained models and utilities.
• PyTorch 2.0.0 and torchvision 0.15.0: Deep learning framework.
• NumPy 1.26.4: Numerical computations.
• Optuna 3.6.1: Hyperparameter optimization.
• XGBoost 2.1.0: Gradient boosting classifier.
• SciPy 1.14.0: Scientific computing.
• Matplotlib 3.9.1: Data visualization.

## Appendix B

Table B.1 lists the best hyperparameters identified for the XGBoost classifier during hyperparameter tuning. These values represent the optimal settings for the classifier based on cross-validation performance on the TCGA dataset.

## Footnotes

1 The use of the UNI model in our study was discarded at an earlier stage in favor of the Prov-GigaPath model for further improvements. Consequently, there is no further information on the UNI model's performance in our study.
